# Human papillomavirus infection and disease recurrence/persistence after treatment for women of high-grade cervical intraepithelial neoplasia with coexisting vaginal intraepithelial neoplasia

**DOI:** 10.3389/fcimb.2025.1602216

**Published:** 2025-07-09

**Authors:** Jing Zhang, Lina Wu, Yanmei Zhu, Guangcong Liu, Danbo Wang

**Affiliations:** ^1^ Department of Gynecology, Cancer Hospital of China Medical University, Liaoning Cancer Hospital and Institute, Shenyang, Liaoning, China; ^2^ Department of Oncology, Shengjing Hospital of China Medical University, Shenyang, Liaoning, China; ^3^ Department of Pathology, Cancer Hospital of China Medical University, Liaoning Cancer Hospital and Institute, Shenyang, Liaoning, China; ^4^ Department of Epidemiology, Cancer Hospital of China Medical University, Liaoning Cancer Hospital and Institute, Shenyang, Liaoning, China

**Keywords:** high-grade cervical intraepithelial neoplasia, vaginal intraepithelial neoplasia, persistent high-risk human papillomavirus infection, disease recurrence, loop electrosurgical excision procedure

## Abstract

**Background:**

Coexistent cervical intraepithelial neoplasia (CIN) and vaginal intraepithelial neoplasia (VaIN) is problematic, posing challenges for patient management. This study focused on the clinical characteristics of coexistent CIN 2/3 and VaIN (all degrees), evaluating the proclivity for disease recurrence/persistence at 6 months after treatment.

**Methods:**

A retrospective case–control study of women treated for coexistent CIN 2/3 and VaIN (CE group) was undertaken between January 2018 and December 2020. During the same period, women with CIN 2/3 only were selected chronologically (1:2 ratio) for comparison (sCIN group). A loop electrosurgical excision procedure (LEEP) was the standard treatment for CIN 2/3, performing electrofulguration of VaIN in tandem. First follow-up visits at 6 months thereafter entailed testing for human papillomavirus (HPV). Univariate and multivariate analyses served to assess pertinent risk factors.

**Results:**

There were 91 CE group members, each treated for coexistent CIN 2/3 and VaIN (VaIN 1, 35; VaIN 2/3, 56). Age ≥50 years (OR = 3.362, 95% CI: 1.421–7.954) emerged as an independent risk factor for coexistent disease. Positive margins and persistent high-risk HPV (HR-HPV) infection after treatment were more common in the CE (vs. sCIN) group (*p* = 0.012 and *p* < 0.001, respectively), as was recurrent/persistent high-grade disease (17.6% vs. 2.2%; *p* < 0.001). In the CE group, persistent HR-HPV infection 6 months after treatment (OR = 21.320, 95% CI: 2.509–181.188) was the sole independent risk factor for disease recurrence/persistence at 6 months.

**Conclusions:**

Comprehensive vaginal wall examinations are warranted for women with CIN 2/3, particularly those >50 years old. Close follow-up by HPV test is also indicated if CIN 2/3 and VaIN coexist, given a heightened incidence of recurrent/persistent disease.

## Introduction

Cervical cancer is the fourth most common female-related cancer worldwide and represents a significant health problem for women (Sung et al., 2021). Early identification and treatment of cervical intraepithelial neoplasia (CIN) is critical for cervical cancer prevention (Palumbo et al., 2023). High-risk human papillomavirus (HR-HPV) infections, which are linked to precancerous lesions or invasive cancers of the cervix (Bogani et al., 2017), may similarly develop in vaginal and vulvar locations (Bertoli et al., 2019). In recent years, the incidence of vaginal intraepithelial neoplasia (VaIN) has risen (Zhang et al., 2016), with low-grade (VaIN 1) or high-grade (VaIN 2/3) lesions more often detected in the setting of past hysterectomy for cervical cancer or high-grade CIN (CIN 2/3) (Preti et al., 2020). Because VaIN may accompany CIN 2/3 (Perkins et al., 2020), delineating associated clinical characteristics may help alert clinicians to its potential development.

To treat high-grade CIN, a loop electrosurgical excision procedure (LEEP) or cold-knife conization (CKC) is usually recommended (Perkins et al., 2020). However, even if lesions are effectively removed as above (virtually eradicating HPV infection), the risk of recurrence and progression to cervical cancer is higher for such women, compared with others in the general population (Ouh et al., 2020). A recent study suggested that post-treatment HPV vaccination with the nonavalent vaccine (Gardasil 9) may facilitate viral clearance in women treated for HPV-related cervical lesions (Palumbo et al., 2025). However, vaccination uptake remains low in China. In terms of VaIN, the incidence of progression to vaginal squamous cell carcinoma ranges from 2% to 7% (Ratnavelu et al., 2013). Although VaIN 1 is not a preinvasive threat, its rate of spontaneous regression is lower in the presence (vs. absence) of CIN 2/3 or vulvar intraepithelial neoplasia (VIN) 2/3 (67% vs. 91%) (Kesic et al., 2023), and once identified, the premalignant nature of VaIN 2/3 requires intervention. Consequently, all grades of VaIN should be addressed when treating CIN 2/3.

A variety of treatment options have been adopted for managing VaIN, including topical agents, ablation, excision, and radiotherapy (Gurumurthy et al., 2020). Commonly used ablation therapies include CO_2_ laser vaporization, photodynamic therapy, and electrofulguration, the latter being a good choice for VaIN treatment (Chen et al., 2016). The use of the same device for LEEP and electrofulguration is a suitable approach in patients with coexistent CIN 2/3 and VaIN. However, women with VaIN are at high risk of recurrence (Kim et al., 2018). Despite simultaneous treatment, the ramifications of coexistent CIN 2/3 remain unclear in this regard, meriting further research.

The present study was undertaken to investigate the clinical characteristics of coexistent CIN 2/3 and VaIN, analyzing risk factors for disease recurrence/persistence at 6 months after treatment.

## Materials and methods

### Study population

This retrospective, case–control study involved women with histologically confirmed CIN 2/3, each subjected to LEEP between January 2018 and December 2020 at Liaoning Cancer Hospital and Institute of China Medical University. Among them, 91 displayed both CIN 2/3 and VaIN 1 or VaIN 2/3 (CE group), undergoing electrofulguration of vaginal lesions concurrently. Another 182 diagnosed with CIN 2/3 exclusively comprised the single CIN (sCIN) group, having been selected at random according to order of colposcopy-guided biopsy. Exclusion criteria were the following: (1) postoperative pathology upgraded to microinvasive or invasive carcinoma; (2) lack of follow−up data; (3) second conization or total hysterectomy within 3 months after LEEP; (4) conditions likely impacting HPV susceptibility or CIN progression, including autoimmune disorders, genetic disorders, or organ transplantation; and (5) pregnancy. The Ethics Committee of Liaoning Cancer Hospital and Institute approved our study protocol (reference number: 20230162). All subjects were duly informed, granting signed consent before submitting to colposcopy and operative procedures.

### Clinical data

We collected demographic and clinicopathologic data on each patient, recording age at the time of conization, transformation zone type, pre- and postoperative liquid-based cytology (LBC) results, pre- and postoperative HPV infection status, conization outcomes (grade of dysplasia and margins), and follow-up biopsy histopathology. Using ThinPrep technology (Hologic, Marlborough, MA, USA), reporting of LBC results followed the 2014 Bethesda System guidelines. The Digene Hybrid Capture 2 (HC2) test (Qiagen, Hilden, Germany) provided semiquantitative measures of HR-HPV DNA content, equating positivity with a >1 ratio of relative light units to positive control cutpoint (RLU/PC). HPV DNA genotyping was achieved using the HPV GenoArray test kit (Hybribio Ltd, Kowloon Bay, Hong Kong), combining DNA amplification and flow-through hybridization techniques (Liu et al., 2010). Each sample was screened for a total of 21 HPV genotypes (6, 11, 16, 18, 31, 33, 35, 39, 42–45, 51–53, 56, 58, 59, 66, 68, and CP8304).

### Colposcopy and biopsy techniques

Colposcopy was routinely undertaken in our Cervical Disease Clinic, examining the cervix, vagina, and vulva comprehensively. Any suspicious areas were subjected to biopsy under colposcopic guidance, acquiring multiple samples on occasion in areas of patchy involvement or across particularly broad lesions to exclude occult invasion. Colposcopic examinations were deferred if needed, pending treatment of cervical/vaginal infections or postmenopausal dystrophy (i.e., estrogenic therapy). Within the sCIN group, we biopsied vaginal wall at points unstained by Lugol’s iodine where VaIN might reside. Histologic sections of routinely processed tissues were independently assessed by two expert pathologists.

### Operative procedures

All LEEP and electrofulguration operations were performed under the guidance of colposcopy. In subsequent pathologic examinations of cone specimens (marked at 12 o’clock), positive margins were those harboring CIN 1–3 or cancer at one or both resected edges (ectocervical or endocervical). We set the power rating of electrofulguration output to 30 W, placing the head of the ball electrode close to its surface target. Treatment range was 3 mm beyond the outer edge of lesion, at a depth of 1.0–1.5 mm.

### Follow-up monitoring

Study enrollees regularly returned for first follow-up visits 6 months post-treatment, at which time each supplied samples for HC2 HPV DNA testing and LBC. Detectable abnormalities called for colposcopy-directed biopsy of the cervix or endocervical curettage. Disease recurrence/persistence was signaled by histologically confirmed CIN 2/3, VaIN 2/3, or both on post-treatment biopsies at 6 months.

### Statistical analysis

We expressed non-normally distributed quantitative variables as median values and interquartile ranges (IQRs), using the Mann–Whitney *U* test to compare differences. Categorical data were shown as counts and percentages. To compare groups, Pearson’s *χ* ^2^ test was employed, applying Fisher’s exact test to small-sized samplings. Logistic regression served to generate odds ratios (ORs) and 95% confidence intervals (CIs). Independent risk factors were identified through multivariate logistic regression. All computations were driven by standard software (SPSS v22.0; IBM Corp, Armonk, NY, USA), with significance set at *p* < 0.05.

## Results

### Population characteristics

A total of 91 women qualified for the CE group, demonstrating coexistent CIN 2/3 and VaIN (VaIN 1, 35; VaIN 2/3, 56). In the sCIN group, there were 182 women with CIN 2/3 only. The clinical characteristics of the study population are shown by group in **Table 1**. Median age in the CE group (45 years, IQR: 36–52; range 21–68 years) significantly surpassed that determined for the sCIN group (38.5 years, IQR: 32–45; range, 22–62 years; *p* < 0.001), with significantly more women ≥50 years old by comparison (37.4% vs. 11.5%; *p* < 0.001). Results of HPV genotyping, which were available in part (CE group, 56; sCIN group, 140), showed similarity in terms of HPV16/18 infection prevalence (62.5% vs. 60.0%; *p* = 0.746). However, multiple HPV infections were significantly more common in the CE (vs. sCIN) group (44.6% vs. 29.3%; *p* = 0.04), with type III cervical transformation zones predominating (61.5% vs. 38.5%; *p* < 0.001). VaIN found in CE group members initially was largely multifocal (63/91, 69.2%), far less inclined to unifocal involvement (28/91, 30.8%). VaIN lesions were confined to vaginal fornices or the upper one-thirds of vaginal canals in a majority (75/91, 82.4%) of women.

**Table 1:** Characteristics and comparisons of women in the CE group and the sCIN group.

Characteristics	CE group (*n* = 91)	sCIN group (*n* = 182)	Univariate		Multivariate	
			*χ* ^2^	*p*	OR (95% CI)	*p*
Age, median (IQR)	45 (36, 52)	38.5 (32, 45)		<0.001		
<50, *n* (%)	57 (62.6)	161 (88.5)	25.148	<0.001		
≥50, *n* (%)	34 (37.4)	21 (11.5)			3.362 (1.421–7.954)	0.006
Cytology pre-operation *						
NILM, n (%)	17 (23.3)	45 (24.7)	0.059	0.809		
ASC-US or worse, n (%)	56 (76.7)	137 (75.3)				
HPV infection pre-operation						
Negative, n (%)	4 (4.4)	4 (2.2)		0.447		
Positive, n (%)	87 (95.6)	178 (97.8)				
HPV genotype †						
Non-HPV16/18, n (%)	21 (37.5)	56 (40.0)	0.105	0.746		
HPV16/18, n (%)	35 (62.5)	84 (60.0)			0.790 (0.399–1.563)	0.498
Multiple HPV infection †						
No, n (%)	31 (55.4)	99 (70.7)	4.224	0.040		
Yes, n (%)	25 (44.6)	41 (29.3)			1.860 (0.950–3.643)	0.070
Type of transformation zone						
Type I, n (%)	9 (9.9)	31 (17.0)				
Type II, n (%)	26 (28.6)	81 (44.5)			1.832 (0.560–5.994)	0.317
Type III, n (%)	56 (61.5)	70 (38.5)	13.0	<0.001	2.345 (0.696–7.897)	0.169
Number of VaIN lesions						
Multifocal, n (%)	63 (69.2)					
Unifocal, n (%)	28 (30.8)					
Localization of VaIN lesions						
Vaginal fornices, n (%)	21 (23.1)					
Upper third, n (%)	54 (59.3)					
Middle third, n (%)	13 (14.3)					
Lower third, n (%)	3 (3.3)					

* There were 73 women in the CE group with available cytology results pre-operation.

† There were 56 women in the CE group and 140 women in the sCIN group with available HPV genotyping results pre-operation.

CIN, cervical intraepithelial neoplasia; VaIN, vaginal intraepithelial neoplasia; HPV, human papillomavirus; OR, odds ratio; CI, confidence interval; NILM, negative for intraepithelial lesion or malignancy; ASC-US, atypical squamous cell of undetermined significance.

Multivariate analysis succeeded in identifying age ≥50 years (OR = 3.362, 95% CI: 1.421–7.954) as the sole independent risk factor for coexistent CIN 2/3 and VaIN, failing to implicate HPV genotype, multiple HPV infections, or type III transformation zone.

### Disease recurrence/persistence 6 months after treatment

Women of both groups presented for first follow-up visits 6 months after treatment. Clinicopathologic characteristics following therapy are listed by group in **Table 2**. The margin positivity rate was significantly higher for the CE (vs. sCIN) group (33.0% vs. 19.2%; *p* = 0.012). At this time, persistent HR-HPV infection was also significantly more common in CE (vs. sCIN) group members (48.4% vs. 23.1%; *p* < 0.001), mirrored by the corresponding rates of recurrent/persistent high-grade disease [17.6% (16/91) vs. 2.2% (4/182); *p* < 0.001]. However, there were no instances of progression to invasive cancer. In the CE group, 12 women experienced recurrent/persistent disease of both cervix and vagina, whereas vaginal lesions alone were noted in four others. In contrast, recurrent/persistent lesions of the cervix only presented in three women of the sCIN group, with one exhibiting both cervical and vaginal lesions.

**Table 2:** Comparisons of the clinicopathological factors after treatment between the CE group and the sCIN group.

Characteristics	CE group (*n* = 91)	sCIN group (*n* = 182)	*χ* ^2^	*p*
Cervical histologic result post-operation, *n* (%)				
CIN2/3	52 (57.1)	101 (55.5)	0.067	0.796
CIN1 or inflammation	39 (42.9)	81 (44.5)		
Margin status, *n* (%)				
Positive	30 (33.0)	35 (19.2)	6.310	0.012
Negative	61 (67.0)	147 (80.8)		
Cytology results at 6 months after treatment, *n* (%)				
Abnormal	23 (25.3)	20 (11.0)	9.330	0.002
Normal	68 (74.7)	162 (89.0)		
HPV infection at 6 months after treatment, *n* (%)				
Positive	44 (48.4)	42 (23.1)	17.960	<0.001
Negative	47 (51.6)	140 (76.9)		
Recurrent/persistent disease at 6 months after treatment, *n* (%)				
Yes	16 (17.6)	4 (2.2)	21.149	<0.001
No	75 (82.4)	178 (97.8)		

CIN, cervical intraepithelial neoplasia; HPV, human papillomavirus.

### Risk factors for disease recurrence/persistence 6 months after treatment (CE group)

In univariate logistic regression analysis, persistent HR-HPV infection 6 months after treatment (OR = 23.793, 95% CI: 2.982–189.862) emerged as the sole variable associated with post-treatment disease recurrence/persistence (**Table 3**). Age, a multiplicity of preoperative HPV infections, HPV genotype, margin status, preoperative grade of coexistent VaIN, and preoperative multi- or unifocal VaIN were unrelated factors.

**Table 3:** Risk factors associated with recurrent/persistent disease after treatment in the CE group.

Characteristics	Total	Residual/recurrent disease, *n* (%)	Univariate		Multivariate	
			OR (95% CI)	*p*	OR (95% CI)	*p*
Age						
<50, n (%)	57	7 (12.3)				
≥50, n (%)	34	9 (26.5)	2.571 (0.857–7.711)	0.092	1.894 (0.529–6.781)	0.326
Preoperative multiple HPV infection *						
No, n (%)	31	7 (22.6)				
Yes, n (%)	25	6 (24.0)	0.924 (0.266–3.209)	0.900		
Preoperative HPV genotype *						
Non-HPV16/18, n (%)	21	5 (23.8)				
HPV16/18, n (%)	35	8 (22.9)	0.948 (0.264–3.400)	0.935		
Margin status						
Negative, n (%)	61	9 (14.8)				
Positive, n (%)	30	7 (23.3)	1.758 (0.584–5.298)	0.316	3.018 (0.756–12.049)	0.118
Preoperative grade of coexistent VaIN						
VaIN1, n (%)	35	8 (22.9)				
VaIN2/3, n (%)	56	8 (14.3)	0.563 (0.190–1.669)	0.300	0.653 (0.181–2.357)	0.516
Preoperative multifocal VaIN						
No, n (%)	28	2 (7.1)				
Yes, n (%)	63	14 (22.2)	3.714 (0.784–17.606)	0.098	4.265 (0.685–26.562)	0.120
Persistent HR-HPV infection at 6 months after treatment						
No, n (%)	47	1 (2.1)				
Yes, n (%)	44	15 (34.1)	23.793 (2.982–189.862)	0.003	21.320 (2.509–181.188)	0.005

* There were 56 women in the coexistent group with available HPV genotyping results pre-operation.

VaIN, vaginal intraepithelial neoplasia; HPV, human papillomavirus; HR-HPV, high-risk human papillomavirus; OR, odds ratio; CI, confidence interval; NILM, negative for intraepithelial lesion or malignancy; ASC-US, atypical squamous cell of undetermined significance.

Multivariate analysis was carried out as well, adjusting for potential confounding effects (i.e., age, margin status, preoperative grade of coexistent VaIN, and preoperative multi- or unifocal VaIN). The only risk factor identified for post-treatment disease recurrence/persistence was HR-HPV infection 6 months after treatment (OR = 21.320, 95% CI: 2.509–181.188) (**Table 3**).

## Discussion

Results of the present study confirm that the coexistence of CIN 2/3 and VaIN is a rather common event. The one related risk factor that emerged in the course of our analysis was age ≥50 years. During follow-up, a poorer prognosis was evident for the CE group by comparison, having shown an increased rate of margin positivity, greater persistence of HR-HPV infection, and higher risk of recurrent/persistent disease 6 months after treatment. In fact, persistent HR-HPV infection 6 months after treatment proved to be an independent risk factor for disease recurrence/persistence at 6 months in the CE group. Compared with the sCIN group, CE group members were more prone to recurrent/persistent vaginal wall lesions.

In the past, hysterectomy due to CIN 2/3 and stage I cervical cancer has been the chief issue driving the clinical awareness of VaIN. However, it is now apparent that CIN and VaIN often coexist, reported at rates of 2.2%–15.7% in previous studies (Zhang et al., 2016; Gonzalez-Bosquet et al., 2017) and increasingly raising concerns. Previous studies have linked coexisting CIN and VaIN to older age, multiple HPV infections, high HPV viral load, HPV16 infection, and immunosuppression (Gonzalez-Bosquet et al., 2017; Zhang et al., 2022; Cho et al., 2021). Consistent with these findings, our study identified age ≥50 years and multiple HPV infections as risk factors; however, only age ≥50 years remained an independent predictor. This association may reflect reduced estrogen levels and age-related immunologic decline. Thinning of the vaginal epithelium in older women may increase vulnerability to microtrauma, thereby facilitating HPV infection and impairing viral clearance (Zhang et al., 2024).

Vaginal colposcopy poses greater challenges than cervical colposcopy due to the nonspecific and variable appearance of VaIN on colposcopic examination (Sopracordevole et al., 2018). Notably, occult invasive carcinoma has been detected in 2.6%–5.5% of cases (Chai et al., 2020; Sopracordevole et al., 2020), underscoring the importance of obtaining multiple biopsies, particularly from areas with the most suspicious colposcopic features.

The management of VaIN remains challenging due to the proximity of adjacent organs such as the bladder and bowel and the complexity of treating multifocal or extensive lesions (Gurumurthy et al., 2020). While surgical excision may be preferred, its utility is limited by the often patchy and multifocal nature of VaIN. In our cohort, multifocal VaIN was present in 69.2% of women with coexistent disease. Alternatively, ablation is considered effective for VaIN 2/3, especially for multifocal lesions, and is associated with low complication rates (Cho et al., 2021; Sopracordevole et al., 2020). Both ablative and excisional approaches have demonstrated comparable efficacy (Bogani et al., 2018). Furthermore, the equipment used for LEEP can also be applied to electrofulguration, which has proven to be a safe and effective treatment with favorable cure rates and minimal complications (Kesic et al., 2023).

Ahead of ablative procedures, lesions should be fully visible and adequately biopsied to exclude invasion. VaIN 1 in the presence (vs. absence) of CIN 2/3 carries a lower spontaneous regression rate due to the different biologic behavior (Kesic et al., 2023). In our study population, we treated multiple foci of VaIN 1 and CIN 2/3 concurrently to achieve full eradication and reduce the subsequent recurrent/persistent disease rate in CE group members. A prospective study is needed going forward to better assess the benefit of this approach.

Even if lesions are effectively removed by various procedures, such as resection or ablation, some risk of persistent HR-HPV infection remains post-treatment in affected patients. Several earlier studies examining HR-HPV status following LEEP for CIN 2/3 have cited rates of persistent HPV at 6 months in the range of 14.3%–33.0% (Kim et al., 2010; Pirtea et al., 2016). The testing we required during first follow-up visits (6 months post-treatment) confirmed a significantly higher rate of persistent HR-HPV infection in the CE (vs. sCIN) group (48.4% vs. 23.1%; *p* < 0.001). This outcome perhaps reflects a pathogenesis inherent in multicentric lesions of lower genital tract that is marked by divergent cervical and vaginal proclivities for specific HPV genotypic infections (Zhang et al., 2021). Currently, overall rates reported in the literature for CIN 2+ recurrences after LEEP range from 5% to 25% (Ouh et al., 2020; Fernández-Montolí et al., 2020). Recurrences of VaIN are also quite frequent (10% to 42%), despite standard treatment (Field et al., 2020; Dodge et al., 2001), and recurrence rates for multicentric lesions tend to exceed those observed for isolated CIN or VaIN involvement (Ait Menguellet et al., 2007). In our hands, the rate of high-grade lesion recurrence/persistence in CE group members was 17.6% during follow-up, readily surpassing that of the sCIN group (2.2%; *p* < 0.001). Hence, it is our view that women with coexistent CIN 2/3 and VaIN should be closely monitored during post-treatment periods.

Predicting recurrence expeditiously after surgery is vital. Recurrence rates are ostensibly influenced by menopause, lesion size, smoking habit, CIN detected at margins, HIV positivity, and persistent HR-HPV infection (Bogani et al., 2018; Wu et al., 2016; Jing et al., 2018; Ricci et al., 2025). Although margin status is a convenient predictor of recurrence and a customary risk marker, it is not an acknowledged independent risk factor (Massad et al., 2013). HR-HPV testing has instead assumed a more critical role in postoperative follow-up (Ryu et al., 2012; Bruhn et al., 2022). The 2019 guidelines of the American Society of Colposcopy and Cervical Pathology (ASCCP) recommended HPV-based testing at first follow-up (preferably 6 months after treatment of high-grade CIN), regardless of margin status (Perkins et al., 2020). There is mounting evidence that HR-HPV persistence is pivotal in predicting recurrence after conization for CIN 2/3 (Bogani et al., 2018; Gosvig et al., 2015; Du et al., 2013). Unfortunately, no data are available as yet to support this premise in the context here. Our findings with respect to the CE group seem to corroborate HR-HPV persistence as an independent risk factor for disease recurrence/persistence. Multifocality is a notable characteristic of VaIN. A recent study identified multifocality as an independent risk factor for recurrence or persistence of VaIN following laser vaporization (Boonlikit and Tangterdchanakit, 2024). However, in the present study, multifocality was not significantly associated with recurrence or persistence (OR = 3.714, 95% CI: 0.784–17.606). Further research into broader populations is needed for validation.

Ultimately, we have demonstrated that women with coexistent CIN 2/3 and VaIN are more apt to experience vaginal wall recurrence/persistence. In the sCIN group, a solitary instance of post-treatment coexistent disease was apparent. It is feasible that this specific virus was latent within the vaginal wall, underscoring the need for full examinations during follow-up colposcopy.

The main strengths of this study include its large roster of enrollees and the substantial volume of comprehensive data, relative to other published reports. Moreover, this study is among the few to examine the clinical characteristics of coexisting CIN 2/3 and VaIN, thereby highlighting the need for increased clinical attention to women presenting with both lesions.

Nevertheless, several limitations must be acknowledged. First, the retrospective and single-center design necessitates cautious interpretation of the findings. Second, because of the lack of a prospective cohort, some confounding variables such as smoking status, immunosuppression, and HPV vaccination status could not be assessed, limiting causal inferences. Third, although HC2 assays are commonly used during follow-up to detect persistent HR-HPV infections, they do not allow genotypic confirmation of viral persistence. Additionally, we were unable to distinguish between cervical and vaginal HPV infections, precluding the assessment of concordance between sites. The short follow-up period, while useful for early observations, limits the ability to draw conclusions regarding long-term recurrence. Notably, HR-HPV infection at 6 months post-treatment (OR = 21.320; 95% CI: 2.509–181.188) was the only identified risk factor for post-treatment disease recurrence/persistence. However, the wide CI reflects substantial uncertainty, likely due to the small number of recurrence/persistence events. Future studies with prospective larger cohorts and extended follow-up are needed to enhance the precision of risk estimates and validate these findings.

## Conclusions

In conclusion, more attention must be devoted to coexistent CIN and VaIN, because an undetected vaginal reservoir may impact HPV clearance rates after treatment. Compared with the sCIN group, the CE group exhibited a significantly higher rate of disease recurrence or persistence. Notably, persistent HR-HPV infection at 6 months post-treatment was identified as an independent risk factor for recurrence/persistence in the CE group. These findings underscore the importance of close post-treatment monitoring using HPV-based testing after LEEP and electrofulguration for coexistent CIN 2/3 and VaIN. Surveillance colposcopy should entail full assessment of both cervix and vagina. Further prospective studies are needed in this specific realm, collecting data long-term to better evaluate the clinical implications of the above risk factors for disease recurrence/persistence.

## Data Availability

The raw data supporting the conclusions of this article will be made available by the authors, without undue reservation.
